# Neuroblastoma versus systemic JIA – a diagnostic dilemma

**DOI:** 10.1186/1546-0096-10-S1-A33

**Published:** 2012-07-13

**Authors:** Lisabeth Scalzi, Greg Hychko, Barbara E Ostrov, Catherine A Bingham, David Ungar, Brandt P Groh

**Affiliations:** 1 Hershey Medical Center, Hershey, PA, USA; 2 Indiana University, Bloomington, IN, USA; 3 Zionsville, PA, USA; 4 Pennsylvania State University/Hershey, Hershey, PA, USA

## Purpose

Children with malignancies, including neuroblastoma (NB), may present to their primary physician with complaints that mimic those seen in systemic juvenile idiopathic arthritis (sJIA). The purpose of this investgation was to identify significant distinctions between patients with systemic JIA and neuroblastoma at the time of disease presentation.

## Methods

A retrospective chart review was completed on all patients less than age 18, between 1990 and 2009 at Pennsylvania State University Children’s Hospital, with a diagnosis of either sJIA or NB. The following variables were identified at the time of presentation; gender, age, race, time to diagnosis, presenting signs and symptoms, neuroblastoma stage, LDH, uric acid, sedimentation rate, CRP, platelet count, hemoglobin, peripheral white blood cell count, percentage of neutrophils and lymphocytes, ferritin, and d-dimer. Other categorical information included whether or not the patients had gait disturbance or extremity pain, fever, rash, abdominal mass, arthritis, fatigue, or weight loss. Individual logistic regression models were evaluated for each variable, with sJIA or NB as the outcome. Given that patients with NB and extremity pain may be referred to orthopedics or rheumatology first, instead of a timely referral to hematology/oncology, a subgroup analysis was completed for those patients.

## Results

There was a total of 65 sJIA and 89 NB patients. Demographically, sJIA patients were significantly older (7.2 vs. 3.0 years of age; p<0.0001). Symptomatically, the sJIA patients had fever, rash,arthritis, and extemity pain or gait disturbance (41% vs. 18%) more frequently (p<0.0001 for all). Laboratories were also significantly different between the groups. NB patients had higher LDH and uric acid levels, while sJIA patients had higher platelet counts, total peripheral WBC, had relative neutrophilia (p<0.0001 for all), and had higher ferritin levels than the NB patients (p=0.001), see Table 1. In the subgroup of 58 children with extremity pain or gait disturbance, 45 (78%) had sJIA and 13 (22%) had NB; see Table 2. NB patients had higher LDH (p=0.01) and lower hgb levels (p=0.003), while sJIA patients had a relative neutrophilia (76% versus 51%; p<0.0001).

**Table 1:** Presentation characteristics of SJIA versus NB patients

Variables	sJIA (65)	Neuroblastoma (89)	
Age (years)	7.2 ± 4.7	3.0 ± 3.5	**<0.0001**

Gender	Famle 51%	Female 42%	0.26
	Male 49%	Male 58%	

Race	White 91%	White 85%	0.86
	Black 6%	Black 7%	
	Hispanic 3%	Hispanic 3%	
		Other 5%	

Fever present (0=no, 1=yes)	45/45 (100%)	12/71 (18%)	**<0.0001**

Time to diagnosis (months)	1.5 (65)	1.2 (78)	0.18

Arthritis	51/65 (78%)	1/71 (1%)	**<0.0001**

Rash	53/64 (83%)	2/71 (3%)	**<0.0001**

Abdominal mass	1/65 (2%)	29/71 (41%)	0.0001

Weight loss	11/65 (17%)	5/71 (7%)	0.07

Gait disturbance or extremity pain	45/65 (41%)	13/73 (18%)	<0.0001

Neuroblastoma stage	n/a	Stage 1 = 19/62 (31%)	n/a
		Stage 2 = 3/62 (5%)	
		Stage 3 = 9/62(14%)	
		Stage 4 =31/62 (50%)	

LDH (units/L)	637 ± 530 (35)	1842 ± 2410 (46)	**0.005**

Uric Acid (mg/dL)	3.3 ± 1.2 (22)	5.1 ± 2.9 (34)	**0.005**

Crp (mg/dL)	14.9 ± 25.3 (26)	10.2 ± 7.2 (4)	0.71

ESR (mm/hr)	81.6 ± 36.3 (58)	61.7 ± 46.0 (13)	0.09

PLT count	464,016 ± 173,148 (62)	327,419 ± 136,488 (62)	**<0.00001**

WBC	18,881 ± 11,449 (63)	10,484 ± 5,892 (68)	**<0.00001**

HGB (g.dL)	10.2 ± 1.80 (62)	10.3 ± 2.46 (67)	0.97

Neurtophil %	73 ± 14 (57)	0.49 ± 0.17 (64)	<0.00001

Lymphocyte %	17 ± 10 (47)	40 ± 17 (64)	**<0.00001**

Ferntin (ng/mL)	4,016 ± 6, 135 (50)	159 ± 205 (29)	**0.001**

D-Dimer elevated	34/36 (94%)	2/2 (100%)	0.73

Aldolase	15.6 ± 11.4 (28)	8.85 ± 1.01 (2)	0.42

**Table 2:** Laboratory values for sJIA and NB subjects with gait disturbance or extremity pain

Laboratory	sJIA	NB	p-value
**LDH**	625 ± 522 (28)	1365 ± 1073 (9)	0.01
**Uric Acid**	3.2 ± 1.1 (20)	4.0 ± 1.2 (8)	0.14
**CRP**	9.9 ± 7.8 (22)	10.2 ± 7.3 (4)	0.95
**ESR**	84 ± 32 (42)	100 ± 32 (6)	0.24
**Hgb**	10.4 ± 1.7 (43)	8.5 ± 2.4 (12)	**0.003**
**Neutrophil %**	76 ± 12 (40)	51 ± 10 (11)	**<0.0001**
**Lympocyte %**	15 ± 9 (40)	37 ± 12 (11)	**<0.0001**
**Ferritin**	4554 ± 6890 (37)	555 ± 135 (3)	0.34
**d-dimer abnormal**	27/20	1/1	0.79
**PTT**	35 ± 6 (30)	31 ± 4 (5)	0.15
**Aldolase**	16 ± 2 (24)	9 ± 8 (2)	0.44

## Conclusion

There are significantly different demographic, presenting symptoms, and laboratories at the time of presentation of patients who are ultimately diagnosed with sJIA or NB. In particular, there are discerning laboratories that may help facilitate an appropriate and timely referal to oncology when extremity pain or gait disturbance are among the intial signs and symptoms.

## Disclosure

Lisabeth Scalzi: None; Greg Hychko: None; Barbara E. Ostrov: None; Catherine A. Bingham: None; David Ungar: None; Brandt P. Groh: None.

